# Scapular Dyskinesis: From Basic Science to Ultimate Treatment

**DOI:** 10.3390/ijerph17082974

**Published:** 2020-04-24

**Authors:** Longo Umile Giuseppe, Risi Ambrogioni Laura, Alessandra Berton, Vincenzo Candela, Carlo Massaroni, Arianna Carnevale, Giovanna Stelitano, Emiliano Schena, Ara Nazarian, Joseph DeAngelis, Vincenzo Denaro

**Affiliations:** 1 Department of Orthopaedic and Trauma Surgery, Campus Bio-Medico University, Via Alvaro del Portillo, Trigoria 200, 00128 Rome, Italy; laura.ambrogioni@gmail.com (R.A.L.); a.berton@unicampus.it (A.B.); v.candela@unicampus.it (V.C.); arianna.carnevale@unicampus.it (A.C.); g.stelitano@unicampus.it (G.S.);; 2 Laboratory of Measurement and Biomedical Instrumentation, Campus Bio-Medico University, Via Alvaro del Portillo 200, 00128 Rome, Italy; c.massaroni@unicampus.it (C.M.); e.schena@unicampus.it (E.S.); 3 Carl J. Shapiro Department of Orthopaedic Surgery and Center for Advanced Orthopaedic Studies, Beth Israel Deaconess Medical Center, Harvard Medical School, Boston, MA 20115, USA; anazaria@bidmc.harvard.edu (A.N.); jpdeange@bidmc.harvard.edu (J.D.)

**Keywords:** rotator cuff, scapula, scapular dyskinesis, shoulder, biomechanics, arthroscopy

## Abstract

*Background*: This study intends to summarize the causes, clinical examination, and treatments of scapular dyskinesis (SD) and to briefly investigate whether alteration can be managed by a precision rehabilitation protocol planned on the basis of features derived from clinical tests. *Methods*: We performed a comprehensive search of PubMed, Cochrane, CINAHL and EMBASE databases using various combinations of the keywords “Rotator cuff”, “Scapula”, “Scapular Dyskinesis”, “Shoulder”, “Biomechanics” and “Arthroscopy”. *Results*: SD incidence is growing in patients with shoulder pathologies, even if it is not a specific injury or directly related to a particular injury. SD can be caused by multiple factors or can be the trigger of shoulder-degenerative pathologies. In both cases, SD results in a protracted scapula with the arm at rest or in motion. *Conclusions*: A clinical evaluation of altered shoulder kinematics is still complicated. Limitations in observing scapular motion are mainly related to the anatomical position and function of the scapula itself and the absence of a tool for quantitative SD clinical assessment. High-quality clinical trials are needed to establish whether there is a possible correlation between SD patterns and the specific findings of shoulder pathologies with altered scapular kinematics.

## 1. Introduction

**Figure 1:**
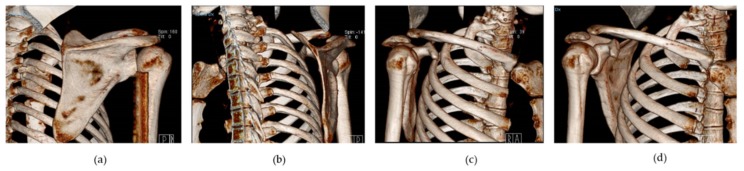
Representation of scapular posterior view (**a**), axillary view (**b**), lateral view (**c**), anterior view (**d**).

The scapula plays a crucial role in coordinating and maintaining complex shoulder kinematics. The rotator cuff (RC) and the scapula control energy and force transfer for glenohumeral (GH) and scapulothoracic (ST) movements [1,2]. From a biomechanical perspective, the shoulder range of motion (ROM) covers almost 65% of a spherical joint whose stability is ensured by several factors such as bone integrity, muscle activity, and ligaments [3]. The RC and scapula allow for three-dimensional movements of the shoulder by limiting excessive translations that may compromise the joint integrity [3,4,5,6]. Patients with shoulder defects (i.e., abnormal 3D GH angulation, subacromial space dimension, GH strain, muscle strength and shoulder muscle activation) often show an alteration of the scapular resting position and motion [4,7,8,9]. In Figure 1, the anatomy of the scapula is represented. 

The condition of abnormal mobility or function of the scapula is called scapular dyskinesis (SD) [10,11]. According to the standard classification, three types of SD can be distinguished: a posterior displacement from the posterior thorax of the inferior medial angle (type I), a posterior displacement from the posterior thorax of the entire medial border of the scapula (type II) and an early scapular elevation or excessive/insufficient scapular upward rotation (dysrhythmia) during dynamic observation (type III) [5,12,13]. 

According to data reported in the literature, SD incidence is frequent in patients with shoulder diseases, including RC diseases, GH instability, impingement syndrome, and labral tears [7,14,15,16,17,18,19,20]. Patients with symptomatic SD show an altered scapular orientation: they present the increasing activity of the serratus anterior, middle and lower trapezius muscles during arm elevation, abduction, and side-lying external rotation (ER) [21]. For these patients, scapular asymmetry in multiple planes is more prevalent during humeral elevation in flexion [22]. Studies demonstrated the occurrence of SD in elite young swimmers, with an incidence of 8.5% on a cohort of 661 asymptomatic elite athletes [23]. Even if asymptomatic, eventually, patients with SD may develop shoulder pain. Patients with shoulder pain have a similar incidence of SD than pain-free subjects [24,25]. An altered scapular upward rotation, especially at 45° and 90° of shoulder abduction, has been observed in overhead athletes who developed the painful type III SD [26]. SD incidence is generally higher in overhead athletes than in non-overhead ones because of the necessity of full upper limb function [27,28,29,30]. Pain could also be affected by musculoskeletal factors, such as a slouched sitting posture. In sedentary young adult males, it has been demonstrated that changing a specific position may influence pain level [31,32]. Altered scapular kinematics could be linked to pain in the region of the neck [33]. An evaluation of muscles in patients with SD and neck pain shows middle trapezius activity during scaption in comparison with neck pain-free patients [34]. In those who complain of SD without pain, altered kinematics does not influence ST muscle activity [34,35]. Despite these findings, clinical evidence to support whether shoulder pain contributes to developing SD or vice versa is still limited. 

SD may be caused or be the cause of shoulder pathologies [36,37,38,39]. In SD, proximal and distal causative factors can be identified. Proximal factors may include the weakness of the scapular muscle, lower trapezius, and serratus anterior, while distal factors may include joint internal imbalance such as labral tears, GH instability, acromioclavicular separation [8]. Proximal factors are usually manageable with rehabilitation, while distal ones need a surgical approach followed by proper rehabilitative protocols [40]. Muscle detachment from the medial border of the scapula leads to SD [41,42]. In young overhead athletes, muscle failure during ER or internal rotation (IR) in abduction could be significant risk factors for shoulder injuries and SD [43,44].

Because of the (still ongoing) broad debate regarding management, treatments, and the causal relationship between shoulder injuries and SD, these issues should be reviewed and evaluated critically. Therefore, the main objective of this study is to summarize the causes and effects, clinical examination and treatments of SD and, secondly, to briefly investigate whether alteration can be managed by a precision rehabilitation protocol planned on the basis of features derived from clinical tests.

## 2. Materials and Methods

A systematic review of the literature was conducted following the Preferred Reported Items for Systematic Review and Meta-Analysis (PRISMA) guidelines [45]. 

A comprehensive search of PubMed, Cochrane, CINAHL and EMBASE databases was performed, using various combinations of the keywords “Rotator cuff”, “Scapula”, “Scapular Dyskinesis”, “Shoulder”, “Biomechanics” and “Arthroscopy”. Three authors (U.G.L., L.R.A., A.B.) performed the search independently. 

The three authors performed the initial title and abstract screening separately, and then the full-text reading, considering the predefined list of inclusion and exclusion criteria. Only articles in English that were published in a peer-reviewed journal were included. Articles were considered eligible for inclusion if they dealt with scapular dyskinesis, its causes and effects, clinical examination, and treatments. Case reports, animal studies, technical notes and letters to editors were excluded. Any disagreements were discussed and resolved by all the authors. Moreover, the reference list of the included studies was manually examined to identify relevant studies not retrieved by the first search. 

**Figure 2:**
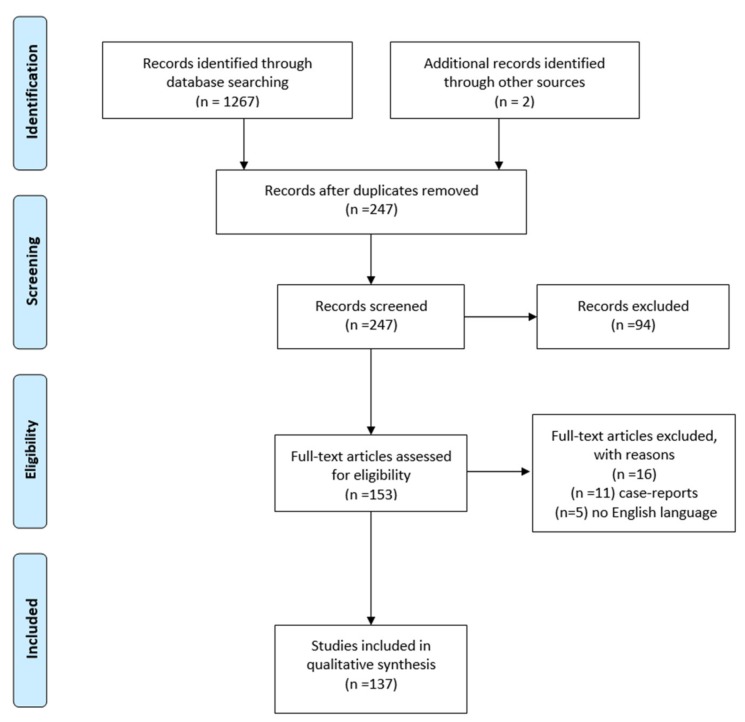
Preferred Reported Items for Systematic Review and Meta-Analysis (PRISMA) 2009 flow diagram.

No meta-analysis was undertaken due to the heterogeneity of the articles. A qualitative description of the included studies was performed. The initial search strategy yielded 1267 articles. After duplicates removal, titles, abstract and full-text screening, a total of 127 studies were included (Figure 2). 

## 3. Causes and Effects of SD

Multiple factors could cause SD, or it could be the cause of shoulder-degenerative pathologies [2]. In both cases, the physical result is a protracted scapula with the arm at rest or in motion [17].

SD could be caused by bony and joint-related issues, neurologic problems, soft tissue problems, and muscle inflexibility. 

Bony issues include thoracic kyphosis that may indirectly promote subacromial impingement syndrome [46,47].

Long thoracic nerve injury may result in medial scapular winging, affecting normal kinematic patterns [17,48,49].

The alteration of soft tissue, such as local inflexibility and muscle stiffness, is the most common reason for abnormal shoulder motion [4,17]. Recently, pectoralis minor stiffness has been investigated, and results demonstrated its role in decreasing the ER and posterior tilt of the scapula during arm elevation [50,51]. Muscle length influences the likelihood of developing SD [52]. In patients with SD, the serratus anterior muscle was less recruited. Clinically, the consequent loss of strength was appreciated by the lesser upward rotation and the greater IR of the scapula [53,54]. Stabilizer muscle fatigue seems to decrease RC strength and to increase SD symptoms [55,56,57]. However, in comparison to patients without SD, no significant difference in ultimate muscle strength was observed, probably because upper trapezius strength can rebalance altered movements [55,58].

Altered scapular orientation is a proven risk factor for impingement, even if it is not the primary cause of disease [59,60]. SD decreases all ROM, apart from forwarding elevation in overhead athletes (baseball, softball, water polo, tennis, racquetball, and volleyball athletes) [17,61,62].

Instability of the acromioclavicular (AC) joint, caused by shortened clavicular malunion, may be the basis of SD [63,64]. The shortening of the clavicle alters scapular kinematics by resulting in a scapular position more anteriorly tilted, upward, and internally rotated position [65]. Moreover, joint imbalance related to the scapula (e.g., high-grade AC joint instability, AC joint arthrosis, GH joint internal imbalance) may affect the kinematic patterns [17,66]. Type III AC dislocation is related to a high incidence of SD, and its surgical reduction, with a hook plate, may decrease SD symptoms [63,64,67,68]. 

When SD is not the primary cause, it could be associated with impingement syndrome, GH instability, clavicular fracture, RC disease, superior labral injury and AC joint pathology, which, in turn, could be related to local tumors, ST crepitus and multidirectional instability [17,69,70,71,72]. Impingement syndrome reduces the scapular ER, and increases upper trapezius muscle activity. Such a syndrome may be caused by the fatigue of RC, leading to the superior migration of the humeral head [59,73]. Like GH instability, impingement affects the optimal relation between glenoid alignment and the muscle length–tension relationship during arm elevation, with a greater loss of shoulder function [14,15,74]. Displaced clavicular fractures make more common the development of painful SD, and they negatively affect clinical outcomes in comparison to subjects without SD [75,76]. Even if not all clavicular fractures are related to altered shoulder motion, SD is present in the long-term after total claviculectomy [77,78]. SD could also be caused by a Latarjet procedure, as demonstrated in five out of 20 patients who undergo this procedure [79]. In other shoulder diseases, such as RC tear, a decreased GH elevation during the abduction, and an increased scapular lateral rotation, are common SD signs [80]. Increasing or maximizing arm elevation may balance the altered RC activation [17,81]. Restored shoulder motion, after RC repair, demonstrates that SD may not always be a trigger of RC tear [80]. Total restored function after superior labral anterior–posterior tears is challenging in comparison to RC tears. SD correction, when caused by labral tears, could need surgery or a conservative approach [82,83,84]. Injuries to the distal segment of the upper limb typically require compensatory movements in the proximal direction, with an indirect influence on scapular motion [85]. Scapular winging and tipping could cause altered kinematics with the overactivity of the middle serratus anterior and the decreased activation of the lower serratus anterior [86,87]. In a cohort of 164 Japanese high school rugby players, stingers represent a causative factor of SD. This suggests that, in addition to the overactivity of the upper limb, a collision may also develop altered scapular kinematics [88]. Therefore, in collision sports, the development of chronic shoulder diseases is not unusual. Such chronic diseases are often linked to SD, so further studies to investigate this relationship and to enhance actual prevention and rehabilitation programs are recommended [89].

## 4. Clinical Examination 

A clinical evaluation of shoulder altered kinematics is complicated due to the difficulty in observing scapular motions alone and the absence of a clinical assessment capable of quantifying SD [22]. Shoulder asymmetry is a recognizable sign during the clinical examination of SD, in both symptomatic and asymptomatic subjects [22]. However, a reliable clinical method to diagnose SD has not yet been developed because of (i) difficulties in observing scapular motions in multiple plans without other muscles and soft tissues and (ii) the absence of clinical assessment compared to a standard for quantifying SD [22]. Some clinical methods have been described with good reproducibility, even though their validity and reliability require further investigations [12,55,90,91,92,93,94,95]. A simple field-based test measuring winging, a lack of control during shoulder motions, and scapula asymmetry, has shown high reliability in musculoskeletal pre-participation screening [96]. Inertial and magnetic measurement systems can be used to assess tri-dimensional kinematic alterations, but their feasibility and validity are not demonstrated yet [97]. The diagnostic accuracy of the Lateral Scapular Slide Test, to identify SD, was found to be poor. [92,98]. The Modified Scapular Assistance Test, using additional handheld weights, may be a reliable clinical method, but its validity has to be confirmed [99]. To measure scapulohumeral translation, a novel technique showed great reliability during scaption and moderate reliability in flexion [100]. To date, evidence-based methods are used to identify abnormalities in shoulder motion patterns [17,74,101,102,103]. From the posterior aspect, the physician focuses on GH and sternoclavicular (SC) joints and evidence of a Scapular Malposition, Inferior Medial Border Prominence, Coracoid Pain and Malposition, and Dyskinesis of scapular movement (SICK position) [17,64]. The combination of visual and palpation methods shows satisfactory inter-reliability for classifying SD [5]. A sitting hand press-up test can be used to evaluate the posterior displacement of the medial border [104]. However, a clinical evaluation seems to be appropriate only in the diagnosis of dyskinesis type I because of a lack of evidence of the effectiveness for type II and III [64,105]. To quantify the features of SD types I and II, a novel scapulometer has been investigated, resulting in an excellent reliability and validity [95]. Furthermore, through plain-film radiography, the coracoid upward shift distance and length of the scapular spine line could be measured; differences in each parameter between the two sides can be correlated to type I and type II of SD, respectively [106]. In the type I subject, a correlation between the simultaneous activation of the middle and lower trapezius muscles, posterior tip, and the upward rotation of the scapula has been demonstrated [107]. In type II, the simultaneous activation of the serratus anterior, upper and middle trapezius muscles has been found [107], without any correlation to scapular ER.

Because of the dynamic components of most shoulder pathologies that result in SD, clinical examination is preferred to static imaging techniques [2]. In baseball players with and without Bennett lesions, further limitations of imaging techniques and clinical examinations exist due to the inability to differentiate between pathologic lesions [108]. However, imaging techniques may be a synergistic component of a precise diagnosis of SD etiology. CT scans, particularly four-dimensional CT scans, allow for the investigation of scapular position and soft tissue to assess the cause of symptoms [109]. Differentially, MRI scans can identify inflammation or lesions that suggest an alteration of the scapular kinematics [110,111]. A good screening tool for the presence of SD is the yes/no method that categorizes abnormal shoulder types I, II, and III of SD into the “yes” category and type IV into the “no” category [22]. Therefore, four-type classification, yes/no classification and an SD test can be considered valuable methods that provide SD evaluation [112]. However, they do not seem to be useful in differentiating between shoulder pathologies, because of the sensitivities of 71% and 41% for the SD and SICK scapula tests, respectively [113]. In patients with subacromial impingement syndrome, the SD test does not measure functional impairment and outcomes in patients with or without SD [114]. Furthermore, in SD tests, a muscle adaption in healthy overhead athletes who may develop shoulder pathologies has been discovered [115,116]. The SD test gave negative results in winged or tipped scapula after two months of rehabilitation and performing dual-wall push-up plus exercises [117]. The index of levator scapulae measures levator scapular muscle strength, and it is reliable in subjects with scapular downward rotation syndrome, rather than in those without this syndrome [118]. Furthermore, strength can be measured in terms of functional outcomes, meaning that the Hole Peg Test is preferred over the Western Ontario Rotator Cuff Index [119]. The infraspinatus strength test showed good reliability to assess infraspinatus weakness due to SD [120]. In patients with trapezius myalgia and SD, the weakness or dysfunction of the scapula-stabilizing muscles do not show differences between healthy patients in terms of winging, delayed movement start, and active proprioception/reposition [121]. An analysis of the ROM and a tridimensional kinematic analysis should be included in clinical assessment because measurements are meaningful in subjects with shoulder pathologies and SD [122]. However, no reliable results were found using electromagnetic tracking to record tridimensional scapular kinematics in patients with or without impingement symptoms [123]. Computerizing shoulder motion may be an effective method to evaluate SD [77]. To investigate ST movements, a regressive approach was developed. Such a method considers the acromion process position, scapula, and humerus orientation compared to the trunk and the relative orientation between the humerus and trunk, and it is able to predict their variation during motion [124]. Test positions for SD assessment showed effective results [12]. In swimmers, the higher prevalence of SD may be explained by the fatigue of muscles that have to stabilize the scapula. In this case, measuring SD post-training could be adequate [125]. The fatigue condition in male tennis players produces an asymmetry in the upward scapular rotation instantly, but this condition does not persist over time [126]. In tennis players, forehand drives, linked to scapular anterior tilt and IR, may contribute to SD [127]. In athletes with reduced SD subacromial space, the occurrence of dynamic disbalance is higher [128]. Therefore, SD evaluation should be included in the differential diagnosis of shoulder pain in competitive swimmers in order to achieve an optimal treatment plan and to accelerate their return to competition [129]. In subjects with RC tear, corrective maneuvers (i.e., scapular assistance test and scapular retraction test) aim to evaluate muscle performance. A scapular assistance test can be used to assess the presence of excessive anterior scapular tilt. It is useful for verifying external impingement symptoms and, by increasing acromiohumeral distance, for identifying subacromial compression [130]. 

## 5. Treatment 

Treatment of SD, both conservative and surgical, aims to restore the scapular position and dynamics [131,132]. 

Conservative treatment in SD cases aims to restore scapular retraction, posterior tilt, and ER. Specific exercises for scapular rehabilitation include flexibility exercises to decrease scapular traction, and scapular stabilization exercises to optimize scapular kinematics. 

**Figure 3:**
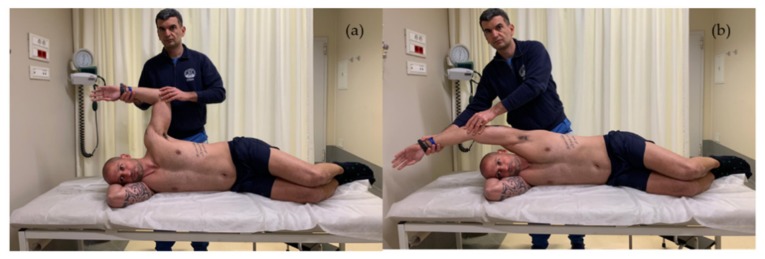
Shoulder horizontal abduction stretching at 90° (**a**) and 150° (**b**).

The traction on scapular posture can be reduced by performing exercises that increase muscle flexibility [50,133]. Stretching exercises with shoulder horizontal abduction at 90° and 150° of elevation have been demonstrated to be useful in increasing pectoralis minor flexibility and the ER and posterior tilt of the scapula during forward elevation [50,133,134] (Figure 3).

**Figure 4:**
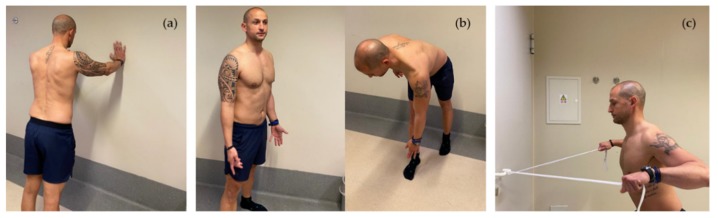
Representation of push up exercises (**a**), lawnmower exercises (**b**), and resisted scapular retraction (**c**).

Scapular stabilization exercises, based on stretching and strengthening, aim to improve muscle strength and joint position sense [26,135,136]. The serratus anterior and trapezius muscles act as scapular stabilizers. The serratus anterior plays an essential role in determining scapular ER and posterior tilt, and the lower trapezius helps to stabilize the scapular position. Scapular stabilization exercises are based on closed and open kinetic chain exercises, including push-ups on a stable or unstable surface, lawnmower exercises and resisted scapular retraction [26,136] (Figure 4).

Push-ups on a stable surface improve the serratus anterior stretch and, when Red Cord slings are used, general muscle strength improvements are obtained [137]; the same exercise, performed on an unstable surface, increases the activation of the trapezius, while decreasing the activation of the serratus anterior [138]. The upper and lower trapezius muscles can be better stimulated with upward rotation shrugs [139]. Specific shrug exercises may be beneficial to increase the upward rotation angle and the upper trapezius activity in subjects with SD and the corresponding scapular downward rotation syndrome [140]. 

A randomized trial showed that exercises for SD with electrical stimulation, performed at 120° of shoulder abduction, improve the distance between the spine and scapula [141]. 

In patients with chronic type III AC dislocations (causes of SD and SICK), functional outcomes improve the performance of strengthening and stretching exercises of the scapular muscles [142,143]. The effects of the Kinesio taping method have also been evaluated. For type II SD, the placement of Kinesio taping over the upper and lower trapezius muscles may rebalance the scapular muscles, increasing the upward scapular rotation [144]; on the other hand, they do not induce changes in the electromyographic activity of serratus anterior, upper and lower trapezius muscles. No alteration in isometric force during shoulder flexo-abduction and external rotation has been shown [145]. Patients with SD can also suffer from neck pain; a randomized clinical trial showed that global postural re-education, aimed at stretching the posterior and anterior muscular chains, compared with conventional stretching exercises, improves patients’ quality of life and pain [33]. 

Conservative therapy is a useful treatment for posterior shoulder injuries associated with SD, such as subacromial impingement syndrome, and it should be focused on restoring RC and posterior capsula function [146,147,148,149]. The first attempt in the conservative treatment of subacromial impingement syndrome, associated with SD, may consist of local infiltration. However, the infiltration of subacromial anesthetics was not effective in restoring the symmetry of scapular kinematics [150]. 

Non-operative treatments may be advantageous for athletes who continuously practice overhead activities. In overhead athletes (e.g., baseball pitchers), the shoulder joint is predisposed to incur altered configurations of the GH joint, ROM deficits, and muscle weakness, resulting in an SD whose grade of injury increases with the level of the competition [151,152,153,154,155,156]. In elite athletes with common internal impingement, researchers have found that treatments focused on intense non-operative approaches provide better outcomes than other treatments and that physical training protocols might be integrated into their usual daily exercises [157,158,159]. Physical training protocol should promote the reinforcement of the scapular muscles and guarantee the optimal length–tension relationship of RC muscles [160]. The exercise programs include the neutralization of scapular positions and the strengthening of scapular stabilizers, i.e., the lower and medium trapezius, serratus anterior, and rhomboids [160,161,162].

The Oslo Sports Trauma Research Center Shoulder Injury Prevention Programme includes exercises aimed at improving the kinetic chain and thoracic mobility. On 660 elite handball athletes, this protocol reduced the prevalence of shoulder problems [163]. Therefore, a prevention program for handball athletes should be based on the improvement of the scapula and GH joint in terms of ROM, control, and strength [164]. Because of the variety and rapidity of shoulder changes, overhead athletes should be continuously monitored during their competition season [165]. Rehabilitation and/or prevention protocols for swimmers should include such exercises as cross-training and core endurance training aimed at stretching the posterior muscles and pectoralis in order to reduce exposure and gain strength [166]. However, in the same class of athletes, training has been found to induce SD in previously pain-free swimmers [155,156,167]. For tennis players with internal impingement, rehabilitation programs should integrate kinetic chain training from the initial phases; angular and translational mobilizations should be carried out to reacquire IR and to posteriorly stretch the GH joint. If SD is present, strengthening exercises of the retractors, aimed at rebalancing the scapula and RC stabilization muscles, should be included [168]. Functional outcome derived from conservative treatment may be predicted by a psychomotor skills test and, thus, change the management of SD [169]. These results show that the large variety of SD alterations could be managed only by a precise rehabilitation protocol planned using individual features derived from clinical and isokinetic tests [170]. 

When a conservative approach fails or joint internal damages occur (e.g., AC separation, GH injuries, the detachment of scapular muscles), surgical treatment should be taken into consideration [17,171]. AC and coracoclavicular ligament disruption changes scapular and clavicular kinematics. Only through surgical reconstruction is the restoration of biomechanical stability possible [172,173,174]. Regarding clavicular injuries, if anterior SC injuries receive non-operative treatment, posterior SC dislocations always need surgery [173]. 

When SD is caused, it is not clear if treatment should focus on the cause or on the altered kinematics. Furthermore, removing the cause does not necessarily result in rebalanced scapular kinematics and vice versa—correcting SD does not always solve the associated shoulder pathology.

## 6. Conclusions

High-quality randomized controlled clinical trials are needed to establish whether there is a possible correlation between SD patterns and specific findings of shoulder pathologies with altered scapular kinematics. Learning to manage SD symptoms may be crucial in the treatment of shoulder diseases and, when SD is the trigger, it could be a fundamental tool to prevent them.
