# Erratum: Longo, U.G., et al. Scapular Dyskinesis: From Basic Science to Ultimate Treatment. *International Journal of Environmental Research and Public Health* 2020, *17(8)*, 2974

**DOI:** 10.3390/ijerph17113810

**Published:** 2020-05-27

**Authors:** Umile Giuseppe Longo, Laura Risi Ambrogioni, Alessandra Berton, Vincenzo Candela, Carlo Massaroni, Arianna Carnevale, Giovanna Stelitano, Emiliano Schena, Ara Nazarian, Joseph DeAngelis, Vincenzo Denaro

**Affiliations:** 1 Department of Orthopaedic and Trauma Surgery, Campus Bio-Medico University, Via Alvaro del Portillo, 200, Trigoria, 00128 Rome, Italy; laura.ambrogioni@gmail.com (L.R.A.); a.berton@unicampus.it (A.B.); v.candela@unicampus.it (V.C.); arianna.carnevale@unicampus.it (A.C.); g.stelitano@unicampus.it (G.S.); denaro@unicampus.it (V.D.); 2 Laboratory of Measurement and Biomedical Instrumentation, Campus Bio-Medico University, Via Alvaro del Portillo, 200, Trigoria, 00128 Rome, Italy; c.massaroni@unicampus.it (C.M.); e.schena@unicampus.it (E.S.); 3 Carl J. Shapiro Department of Orthopaedic Surgery and Center for Advanced Orthopaedic Studies, Beth Israel Deaconess Medical Center, Harvard Medical School, Boston, MA 02215, USA; anazaria@bidmc.harvard.edu (A.N.); jpdeange@bidmc.harvard.edu (J.D.)

The authors would like to correct the names and surnames of the following authors of their previous paper [1], Umile Giuseppe Longo and Laura Risi Ambrogioni.

Therefore, to cite this paper please use the correct reference as follows:

1. Longo, U.G.; Risi Ambrogioni, L.; Berton, A.; Candela, V.; Massaroni, C.; Carnevale, A.; Stelitano, G.; Schena, E.; Nazarian, A.; DeAngelis, J.; et al. Scapular Dyskinesis: From Basic Science to Ultimate Treatment. *Int. J. Environ. Res. Public Health* **2020**, *17*, 2974.
